# Octacalcium Phosphate/Calcium Citrate/Methacrylated Gelatin Composites: Optimization of Photo-Crosslinking Conditions and Osteogenic Potential Evaluation

**DOI:** 10.3390/ijms26146889

**Published:** 2025-07-17

**Authors:** Yuejun Wang, Taishi Yokoi, Masaya Shimabukuro, Masakazu Kawashita

**Affiliations:** 1 Graduate School of Medical and Dental Sciences, Institute of Science Tokyo, 1-5-45 Yushima, Bunkyo-ku, Tokyo 113-8510, Japan; 2 Laboratory for Biomaterials and Bioengineering, Institute of Integrated Research, Institute of Science Tokyo, 2-3-10 Kanda-Surugadai, Chiyoda-ku, Tokyo 101-0062, Japan; shimabukuro.bcr@tmd.ac.jp (M.S.); kawashita.bcr@tmd.ac.jp (M.K.)

**Keywords:** methacrylated gelatin, hydroxyapatite, simulated body fluid, octacalcium phosphate, calcium citrate, gelatin, bone regeneration material

## Abstract

Bone grafting is essential for the regeneration of bone defects where natural healing is inadequate. Octacalcium phosphate (OCP)/calcium citrate (CC)/pig gelatin (pig Gel) composites promote hydroxyapatite (HAp) formation in simulated body fluid (SBF); however, the rapid degradation of pig Gel leads to their degradation in SBF within 7 d. To address this, we developed a 35% OCP/35% CC/30% methacrylated gelatin (GelMA) composite by leveraging the tuneable photo-crosslinking ability of GelMA to enhance the initial structural stability in SBF. However, the optimal synthetic photo-crosslinking conditions and the apatite-forming abilities of the OCP/CC/GelMA composite require investigation. In this study, we employed photo-crosslinking to synthesize homogeneous OCP/CC/GelMA composites with initial structural stability in SBF and evaluated their HAp-forming ability in SBF as an indicator of osteogenic potential, in comparison with the OCP/CC/pig Gel composites. Both GelMA- and pig Gel-based composites were prepared and immersed in SBF for 7 d to assess HAp formation. Although the OCP/CC/GelMA composite showed reduced HAp nucleation compared to the OCP/CC/pig Gel composites, it exhibited enhanced initial structural stability in SBF while retaining its HAp-forming ability. These findings highlight the OCP/CC/GelMA composite as a stable and promising scaffold for bone regeneration, laying the groundwork for further research.

## 1. Introduction

The reconstruction of large bone defects remains a significant challenge in orthopedic surgery because of trauma, tumor resection, and infection [1]. This has driven extensive research into artificial bone substitutes, such as hydroxyapatite (HAp) [2], β-tricalcium phosphate (β-TCP) [3], and octacalcium phosphate (OCP) [4]. These materials are designed to overcome potential immunogenicity and donor site morbidity while mimicking the biological properties of natural bone, including osseointegration and osteoconductivity [5,6].

A previous study revealed that a composite material composed of 35% OCP, 35% CC, and 30% pig gelatin (Gel) demonstrated significant HAp formation in simulated body fluid (SBF) within 7 d, making it a highly promising candidate for bone regeneration applications. One of its components, OCP, a calcium phosphate compound and a precursor to HAp—the primary mineral component of bones—has garnered attention for its remarkable ability to enhance bone regeneration, significantly outperforming hydrolyzed Ca-deficient HAp in supporting osteoblastic activity [7,8,9,10,11,12]. However, the applicability of OCP as a bone substitute is limited by its granular form and chemical structure. The presence of water molecules in its structure may cause loss of its characteristic properties during the sintering process, unlike HAp and β-TCP [13]. To address this, the combination of OCP with diverse synthetic and natural polymeric biomaterials such as collagen, Gel, and poly (lactic-co-glycolic acid) significantly enhances malleability, thereby improving its efficacy as a bone-substitute material [14,15,16,17,18,19]. Among these polymeric biomaterials, Gel, a natural polymer derived from collagen, serves as a biodegradable and biocompatible matrix that is widely used in bone tissue engineering. Its value lies in its role as a scaffold material and its ability to impart osteoconductive properties through OCP crystal grown within a Gel matrix under aqueous conditions. This process yields OCP/Gel composites capable of achieving over 70% new bone formation and exhibiting a biodegradation rate below 3% in a rat critical-sized calvarial defect model, representing 80% of the effectiveness of autogenous bone grafts [15]. Given the benefits of combining OCP with Gel for bone regeneration, other materials have been explored to further enhance the bone regeneration ability of OCP/Gel. Based on the OCP/Gel composite, calcium citrate (CC), a calcium salt with a well-established role in calcium homeostasis, has emerged as a potential biomaterial for bone repair. It can release calcium ions in concentrations that promote osteoblast differentiation and matrix mineralization [20], potentially enhancing osteoblast phenotype progression, biocompatibility, osteoconduction, and osteointegration [21]. Citrate ions play a pivotal role in bone regeneration, as they are essential components of the apatite nanocrystal/collagen complex and contribute to bone formation and mineralization [22]. Exogenous citrate supplementation upregulates the expression of bone development-related genes, such as alkaline phosphatase and osterix, supports the maturation of the osteoblast phenotype, and increases the osteoconductivity of implants [21]. Building on the established benefits of CC in bone repair, its combination with the OCP/Gel composite is expected to synergistically enhance bone regeneration by leveraging its role in bone regeneration.

Although the 35% OCP/35% CC/30% pig Gel composite demonstrated excellent HAp formation in SBF, its rapid degradation within 7 d in SBF for larger samples (e.g., 14 mm diameter) revealed a size-dependent instability, limiting its clinical applicability for bone regeneration [23]. In vivo degradability is a critical factor in bone repair materials, requiring meticulous control. The finding that the degradability varies depending on the size of the pig Gel-based composites is significant. According to Hamada et al., vigorous new bone formation was observed 4 weeks after implantation of the OCP/Gel composite [24], suggesting that synthetic bone substitutes, including OCP/CC/pig Gel, must remain in vivo for at least several weeks.

To address the premature dissolution of the OCP/CC/pig Gel in SBF, we introduced methacrylated gelatin (GelMA), a modified pig-derived Gel with adjustable crosslinking properties, to achieve controlled crosslinking of the composite. By attaching methacrylate groups to Gel, GelMA can undergo photo-crosslinking [25], enabling control over the crosslinking degree. Compared to the thermal dehydration crosslinking used in OCP/CC/pig Gel composites, the photo-crosslinking properties of GelMA offer tunable performance through adjustments in the photoinitiator concentration and light exposure time [25,26]. This controllability enhances the initial structural stability in SBF and effectively addresses the size-dependent instability of the OCP/CC/pig Gel composites, thereby providing a more stable and functionally superior scaffold material for bone tissue engineering. Moreover, the photocuring process of GelMA affords a true hydrogel with a high water content and elasticity, closely mimicking the hydrated microenvironment of native bone matrix. This promotes osteoblast migration, proliferation, and differentiation, thus supporting improved bone regeneration. Furthermore, GelMA facilitates three-dimensional scaffold fabrication and bioprinting, providing a more stable and functionally superior scaffold material for personalized bone tissue engineering [27,28]. Although GelMA has been extensively studied in tissue engineering, typically combined with single calcium phosphate phases (e.g., HAp [29] or β-TCP [30]), the integration of OCP and CC within a GelMA matrix is novel. The aim of this study is to develop a 35% OCP/35% CC/30% GelMA composite that leverages the osteoconductivity of OCP, the mineralization-promoting properties of CC, and the tunable degradation of GelMA to achieve a balance between bioactivity and structural stability, offering a new strategy for designing bone regeneration materials.

However, despite the promising potential of the OCP/CC/GelMA composites for bone tissue engineering, the development and optimization of their synthesis remain largely unexplored. To establish a covalently bonded hydrogel network, GelMA precursors engage in radical polymerization facilitated by a photoinitiator under light of a suitable wavelength and intensity [28]. A promising option is lithium phenyl-2,4,6-trimethylbenzoylphosphinate (LAP), which exhibits maximum absorption at 405 nm. LAP is a single-component initiator with excellent thermal stability, high solubility, and no coloration [31]. Compared with Irgacure 2959, the high water solubility of LAP (8.5 *w*/*v*%) makes it suitable for various crosslinking systems [32]. Notably, Xu et al. demonstrated that LAP-cured scaffolds supported higher cell viability than those cured by Irgacure 2959, indicating the lower cytotoxicity of LAP [33]. Despite being less harmful than UV light, the blue visible light required to activate LAP retains some toxicity [34]. Consequently, in the synthesis of the OCP/CC/GelMA-crosslinked hydrogels, we selected LAP as the photoinitiator to ensure effective crosslinking while limiting the blue light exposure time to minimize potential phototoxicity. This approach aimed to achieve stable hydrogel formation with enhanced biological safety for biomedical applications.

To be considered a promising osteogenic material, OCP/CC/GelMA must be evaluated for its initial bone-forming potential to validate its suitability for bone tissue engineering applications. Understanding the capacity of a material to promote HAp formation is a critical step in assessing its bioactivity and ability to integrate with native bone tissue. The formation of HAp on the surface of a material in SBF serves as a key indicator of its potential to bond with living bone via an HAp layer, as highlighted in a comprehensive review [35]. According to the same review, the degree of HAp formation in SBF can predict the in vivo bone bioactivity of a material, with faster HAp formation corresponding to accelerated bone bonding within the body. Therefore, evaluating HAp formation in SBF is valuable and reliable for predicting the in vivo bioactivity of bone materials prior to animal testing. This approach significantly reduces the number of required animal studies and experimental timelines, thereby accelerating the development of novel bioactive materials.

**Figure 1:**
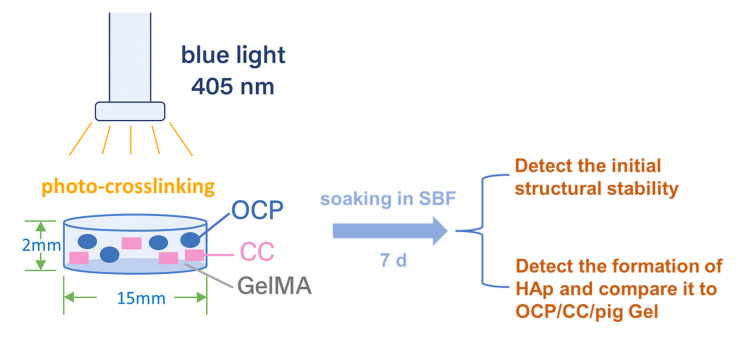
Schematic of the experimental design.

Given the importance of HAp formation as a predictor of bone regeneration potential, it is essential to determine whether replacing pig Gel with GelMA in the OCP/CC composite preserves or alters this property. Previous studies demonstrated strong HAp formation in 35% OCP/35% CC/30% pig Gel composites, with this ratio optimized for maximal HAp formation [23]. Building on these findings, we conducted a comparative evaluation to determine whether the 35% OCP/35% CC/30% GelMA composite, based on the same optimized ratio, retains its ability to form HAp in SBF. To investigate this, we developed a homogeneous 35% OCP/35% CC/30% GelMA composite and immersed it in SBF for 7 d to assess its HAp-forming ability [36], offering insights into its potential as a next-generation bone repair material. The schematic of the experimental design is shown in Figure 1.

## 2. Results

### 2.1. Influence of Photoinitiator Volumetric Ratio and Blue Light Exposure Time on Crosslinking of 35% OCP/35% CC/30% GelMA

The crosslinking behavior of the composite comprising 35% OCP/35% CC/30% GelMA was investigated by varying the volumetric ratio (*v*/*v*) of the LAP photoinitiator and the blue light exposure time. As shown in Table 1, LAP volumetric ratios ranging from 0.02 to 0.1 and exposure times ranging from 1 to 4 min were systematically evaluated to determine their effects on gelation.

At an LAP volumetric ratio of 0.02, no gelation was observed, even after extended exposure for up to 4 min, indicating insufficient photoinitiation to trigger crosslinking. Similarly, at a ratio of 0.06, the composite remained in the liquid state after 1 or 1.5 min of exposure, underscoring the need for a higher LAP volumetric ratio. A significant improvement was observed at an LAP ratio of 0.08, where partial gelation occurred on the upper surface of the composite after 1.5 min of blue light exposure, whereas the bottom part remained liquid. Such a finding suggests that at this ratio, photoinitiation was sufficient to initiate crosslinking near the surface; however, it was inadequate for full-depth gelation. Crosslinking was achieved at an LAP volumetric ratio of 0.1, affording partial gelation (upper surface only) after 1.5 or 2 min of exposure, which progressed to gelation of both the upper and bottom sides after 2.5 min. Under these conditions, no residual liquid phase was detected, indicating robust cross-linking throughout the composite. This level of gelation is critical to ensure the initial structural stability of the hydrogel during subsequent immersion in SBF. Such findings demonstrate that both the LAP volumetric ratio and blue light exposure time synergistically influence the crosslinking efficiency of the 35% OCP/35% CC/30% GelMA composite. Therefore, the optimal conditions for crosslinking were identified as an LAP volumetric ratio of 0.1 with a 2.5 min blue light exposure time; they were determined through extensive experimentation involving multiple trials and the preparation of numerous samples, consistently confirming their reliability and reproducibility. These conditions provide a foundation for further characterization of the properties of the material.

### 2.2. Assessment of Initial Structural Stability of the 35% OCP/35% CC/30% GelMA Composite After Soaking in SBF and Ultrapure Water

**Figure 2:**
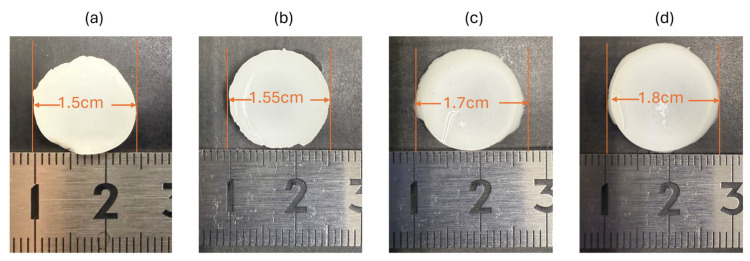
Diameter changes in the 35% OCP/35% CC/30% GelMA sample: (**a**) initial state, (**b**) after 7 d of immersion in SBF, (**c**) after immersion in ultrapure water for 10 min following removal from SBF, and (**d**) after immersion in ultrapure water for 20 min following removal from SBF.

The initial structural stability of the 35% OCP/35% CC/30% GelMA composite was evaluated by measuring dimensional changes under various conditions, as shown in Figure 2. Initially, the composite sample had a circular shape with a diameter of 1.5 cm (Figure 2a). After immersion in SBF at 37 °C for 7 d, the diameter increased slightly to 1.55 cm (Figure 2b), corresponding to an expansion of ~3.3% relative to the initial size. Despite this change, the sample retained its disc shape with no visible signs of dissolution or structural degradation during the 7 d immersion in SBF.

After SBF immersion, the sample was immersed in ultrapure water to remove the remaining PBS and SBF. After 10 min of immersion, the diameter further increased to 1.7 cm (Figure 2c), indicating a 13.3% expansion compared to the initial diameter. Extending the immersion to 20 min resulted in a further increase to 1.8 cm (Figure 2d), equivalent to a 20% expansion from the original size. This progressive swelling behavior suggests a significant water uptake by the GelMA component. Throughout these treatments, the sample exhibited no signs of disintegration or material loss, further confirming that the 35% OCP/35% CC/30% GelMA composite did not undergo early degradation.

These results collectively demonstrate that the 35% OCP/35% CC/30% GelMA composite resists degradation in SBF over 7 d and exhibits swelling behavior in ultrapure water, maintaining structural integrity during the early stages.

### 2.3. Conversion of 35% OCP/35% CC/30% GelMA and 35% OCP/35% CC/30% Pig Gel Immersed in Simulated Body Fluid

**Figure 3:**
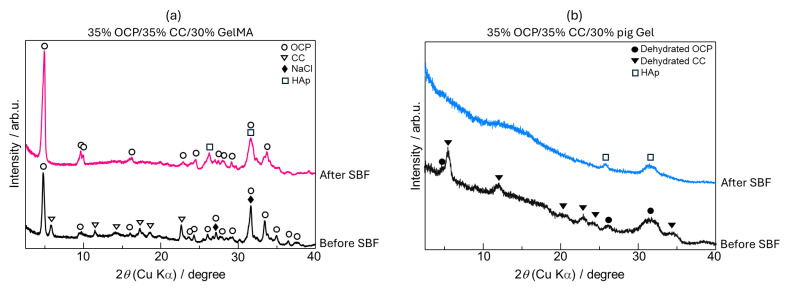
X-ray diffraction (XRD) patterns of (**a**) 35% OCP/35% CC/30% GelMA (15 mm diameter and 2 mm thickness) and (**b**) 35% OCP/35% CC/30% pig Gel (diameter and thickness of 5 mm) before and after immersion in SBF.

**Figure 4:**
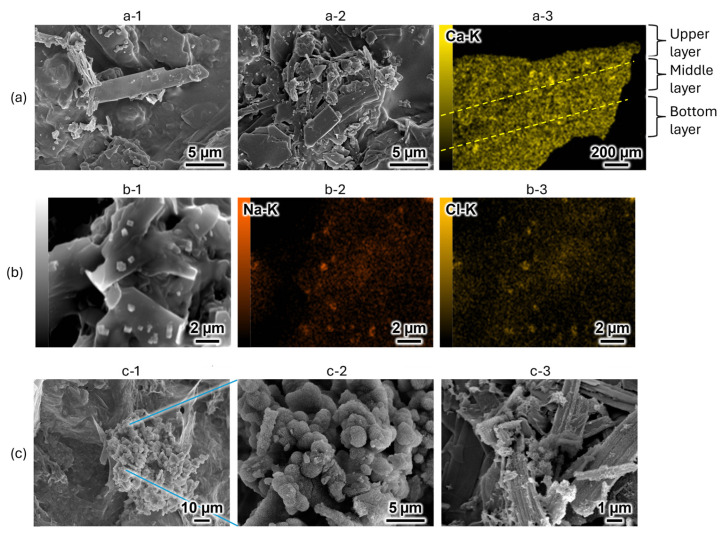
Scanning electron microscopy (SEM) images and energy-dispersive X-ray spectroscopy (EDS) mappings of 35% OCP/35% CC/30% GelMA before and after immersion in SBF. (**a**) 35% OCP/35% CC/30% GelMA before immersion in SBF: (**a-1**) upper side, (**a-2**) bottom side, (**a-3**) cross-sectional EDS mapping of calcium (Ca-K). (**b**) Detailed examination of precipitates on the 35% OCP/35% CC/30% GelMA surface before SBF immersion: (**b-1**) surface structure, (**b-2**,**b-3**) sodium (Na-K) and chlorine (Cl-K) EDS mappings, respectively, of the region in (**b-1**). (**c**) 35% OCP/35% CC/30% GelMA immersed in SBF for 7 d: (**c-1**) surface structure, (**c-2**) magnified image of the central region of (**c-1**), (**c-3**) micrograph of the OCP structure in 35% OCP/35% CC/30% GelMA after SBF immersion.

The X-ray diffraction (XRD) patterns of the 35% OCP/35% CC/30% GelMA sample (15 mm diameter, 2 mm thickness) before and after immersion in SBF for 7 d are shown in Figure 3a. Before SBF immersion, the sample showed the characteristic peaks of OCP, CC, and sodium chloride (NaCl). The presence of NaCl was attributed to the preparation process employing phosphate-buffered saline (PBS), leaving residual NaCl in the composite. After immersion in SBF for 7 d, several notable changes in the crystalline phase were observed, indicating the phase transformation of the 35% OCP/35% CC/30% GelMA composite during SBF soaking, as shown in Figure 3a. 1. The peaks corresponding to CC completely disappeared after SBF soaking, suggesting that CC was either dissolved in SBF or transformed into other phases. 2. The diffraction peaks at 2θ = 26.1° and 31.5°, corresponding to the 002 and 211 reflection peaks of HAp, respectively, exhibited significant broadening after SBF soaking. 3. The intensity of the OCP-specific diffraction peak at 2θ = 4.8° increased after soaking in SBF. 4. The NaCl peaks were no longer present in the XRD patterns of the 35% OCP/35% CC/30% GelMA composite after soaking in SBF followed by washing, as confirmed by the scanning electron microscopy (SEM) images (Figure 4c).

The XRD patterns of the 35% OCP/35% CC/30% pig Gel composite (diameter and thickness of 5 mm), before and after immersion in SBF for 7 d are shown in Figure 3b. Before SBF immersion, the sample displayed diffraction peaks characteristic of OCP and CC after the crosslinking process (heat treatment in vacuum). After 7 d of SBF immersion, the following changes in the crystalline phase were observed. 1. The diffraction peaks of OCP and CC were no longer observed. 2. New diffraction peaks after SBF immersion appeared at 2θ = 25.7° and 31.5°, corresponding to the 002 and 211 reflection peaks of HAp, indicating a complete phase transformation of OCP and CC into HAp during SBF immersion.

SEM was employed to analyze the morphological transformations of the 35% OCP/35% CC/30% GelMA composite before and after immersion in SBF. Before SBF immersion, the material exhibited blade-shaped OCP structures and small fragmented CC particles randomly distributed within the GelMA matrix (Figure 4a-1, upper side; Figure 4a-2, bottom side). To evaluate the homogeneity of the powder distribution (OCP with CC) in the GelMA matrix, cross-sectional analysis was conducted using energy-dispersive X-ray spectroscopy (EDS) (Figure 4a-3). Given that both OCP and CC contain calcium, EDS mapping of calcium (Ca-K) was performed to assess the dispersion of the powder components. The resulting EDS maps revealed a homogeneous calcium distribution across the upper, middle, and lower layers, confirming the homogeneity of the material.

Additionally, SEM imaging before SBF immersion revealed the presence of small, scattered, cuboid-shaped structures (Figure 4b-1) alongside the blade-shaped OCP and fragmented CC. To identify these scattered cuboidal structures, EDS mapping of sodium (Na-K) and chlorine (Cl-K) was conducted in the regions shown in Figure 4b-2,b-3, respectively. The results demonstrated a spatial correlation between the Na and Cl signals and scattered cuboid-shaped structures, confirming that these precipitates were indeed NaCl derived from the PBS solution. These findings correspond to the results of the XRD analysis, revealing the presence of NaCl (Figure 3a, before soaking).

Figure 4c shows the morphology of the 35% OCP/35% CC/30% GelMA composite after immersion in SBF. As shown in Figure 4c-1, part of the material transformed into a cluster-like structure, while magnification of the central region (Figure 4c-2) revealed a dense porous spherical structure. Based on the altered crystalline structure detected in the XRD pattern (Figure 3) and comparison with previously reported SEM images of the HAp structures [23], the cluster-like precipitate in Figure 4c-2 was identified as HAp. This confirms that the 35% OCP/35% CC/30% GelMA composite can form HAp in SBF. Figure 4c-3 presents a micrograph of OCP in the 35% OCP/35% CC/30% GelMA composite after immersion in SBF, revealing small flocculent structures on the surface and surrounding OCP.

By comparing Figure 4b with Figure 4c, it is evident that NaCl is absent after immersion in SBF (Figure 4c). This absence is due to the samples being immersed in ultrapure water after the SBF immersion, which effectively removed NaCl. Such a finding supports the absence of NaCl peaks in the XRD patterns of the SBF-immersed samples in Figure 3a.

**Figure 5:**
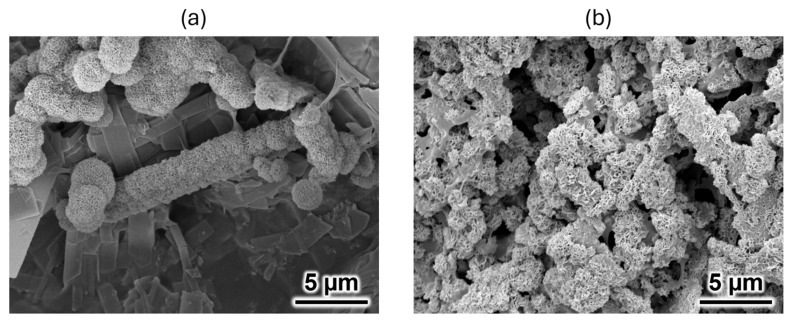
SEM images of the (**a**) 35% OCP/35% CC/30% GelMA and (**b**) 35% OCP/35% CC/30% pig Gel composites after immersion in SBF.

A comparison of the morphologies of the 35% OCP/35% CC/30% pig Gel and 35% OCP/35% CC/30% GelMA composites after immersion in SBF is presented in Figure 5. As shown in Figure 5a, the surface of the 35% OCP/35% CC/30% GelMA composite exhibited spherical and porous HAp structures localized in specific regions. Conversely, the surface of the 35% OCP/35% CC/30% pig Gel composite was almost entirely covered with clustered HAp crystals after SBF immersion (Figure 5b), as confirmed by XRD analysis (Figure 3b). A comparison of Figure 5a,b shows that the surface HAp formation rate in the 35% OCP/35% CC/30% GelMA composite after SBF immersion was lower than that in the 35% OCP/35% CC/30% pig Gel composite.

### 2.4. Comparison of Functional Groups in GelMA and Pig Gel After Crosslinking

To elucidate the differences in functional group structures between GelMA and pig Gel within the composites after crosslinking, pure GelMA and pig Gel samples were characterized using Fourier transform infrared spectroscopy with the attenuated total reflectance (FTIR-ATR) method.

**Figure 6:**
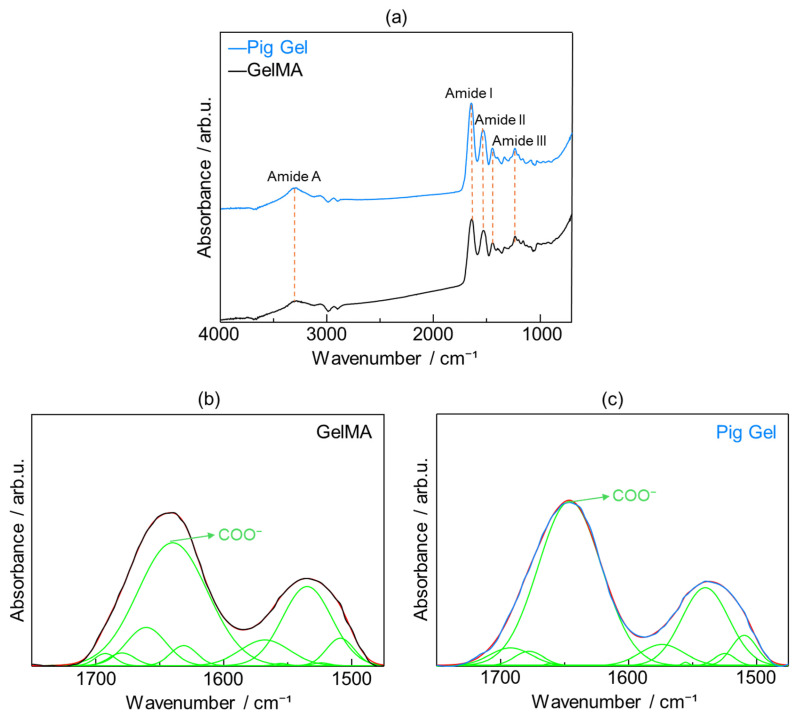
(**a**) Fourier-transform infrared (FTIR) spectra of GelMA (black line) and pig Gel (blue line) after crosslinking; fitting of peaks for (**b**) GelMA and (**c**) pig Gel after crosslinking in the range of 1750–1475 cm^−1^. The original spectra in (**b**) GelMA and (**c**) pig Gel, represented by black and blue lines, respectively, were recovered through cumulative peak fitting (red line), with the individual peaks displayed underneath (green lines).

Figure 6a presents the FTIR spectra of GelMA and pig Gel, highlighting four key spectral regions: 3600–2300 cm^−1^ (Amide A), 1656–1644 cm^−1^ (Amide I), 1560–1335 cm^−1^ (Amide II), and 1240–670 cm^−1^ (Amide III). The Amide A band is associated with N–H stretching and is influenced by hydrogen bonding. The Amide I band is primarily driven by C=O stretching and reflects contributions from hydrogen bonding and COO^−^ interactions. The Amide II band arises from N–H bending and CH_2_ deformations, while the Amide III band is characterized by N–H bending, C–O stretching, and skeletal stretching [37].

Given the critical role of carboxy groups in promoting HAp nucleation in physiological environments [38], the relative abundance of carboxy groups in GelMA and pig Gel was quantified. The Amide I region, dominated by C=O stretching vibrations and modulated by hydrogen bonding and COO^−^ interactions, was selected for detailed analysis. Peak fitting was performed based on previous studies, which identified the following infrared peaks: 1510 cm^−1^ (β-sheet), 1524 cm^−1^ (β-sheet), 1539 cm^−1^ (random coil or helical structures), 1555 cm^−1^ (random coil or helical structures), 1573 cm^−1^ (β-turn), 1618 cm^−1^ carboxy (intermolecular β-sheet), 1630 cm^−1^ (β-sheet), 1645 cm^−1^ (random coil), 1660 cm^−1^ (triple helix), 1677 cm^−1^ (β-turn), and 1692 cm^−1^ (β-turn) [39,40,41,42]. The peak area corresponding to carboxy group-associated vibrations, expressed as a percentage of the total peak area in the 1750–1475 cm^−1^ range, was calculated to be 49.8% in GelMA (Figure 6b) and 60.2% in pig Gel (Figure 6c).

## 3. Discussion

In this study, we identified the optimal conditions for crosslinking the 35% OCP/35% CC/30% GelMA composite hydrogel, achieving gelation of both the upper and lower sides using an LAP photoinitiator ratio of 0.1 (*v*/*v*) and 2.5 min of blue light (405 nm) exposure (Table 1). These conditions represent the minimal LAP concentration and irradiation time required for effective crosslinking, which are essential for maintaining the initial structural stability of the hydrogel in SBF while minimizing potential cytotoxic effects. Previous studies have demonstrated that LAP supports robust cell viability at low concentrations (e.g., 0.3% and 0.5% *w*/*v*) over extended 3D bioprinting durations, such as 60 min [33]. However, cell viability has been observed to decrease with increasing photoinitiator concentration and irradiation time [33]. In our experiments, the LAP concentration was calculated to be 0.15% *w*/*v* based on a 17 mg/mL LAP stock solution and a 0.1 (*v*/*v*) ratio relative to the GelMA solution. This is substantially lower than the 0.3% *w*/*v* concentration reported in previous studies, which achieved 99% cell viability after 15 min of exposure to 365 nm UV light. Moreover, we employed a 405 nm visible light source, which is less damaging to cells than UV light, and a significantly shorter irradiation time of 2.5 min. These milder conditions—lower LAP concentration, shorter exposure time, and less cytotoxic light source—suggest that cell viability is unlikely to be compromised by our crosslinking method. This provides a solid foundation for subsequent cellular experiments to evaluate the impact of the 35% OCP/35% CC/30% GelMA composite on osteoblast proliferation and differentiation. Furthermore, the uncross-linked 35% OCP/35% CC/30% GelMA slurry may hold potential as a bioink component.

XRD and SEM analyses of the 35% OCP/35% CC/30% GelMA and 35% OCP/35% CC/30% pig Gel composites after immersion in SBF, along with FTIR analyses of GelMA and pig Gel after crosslinking, revealed distinct differences in the HAp formation behavior. The XRD patterns of the GelMA-based composite (Figure 3a) did not contain any CC peaks and the HAp peaks at 2θ = 26.1° and 31.5° were broader. Furthermore, the SEM images (Figure 4c) revealed spherical HAp structures with a porous morphology replacing the initially fragmented CC particles, confirming the conversion of CC to HAp. Figure 4c reveals that the blade-shaped OCP structures developed flocculent structures around their edges and on their surfaces, indicating the crystal growth of OCP after immersion in SBF. This observation, coupled with the significant increase in the characteristic OCP peak intensity at 2θ = 4.8° in the XRD pattern (Figure 3a) after SBF immersion, seems to suggest the growth of OCP crystals in the 35% OCP/35% CC/30% GelMA composite after SBF immersion. In contrast, the pig Gel-based composite exhibited complete conversion of both OCP and CC to HAp. This was evidenced by the absence of OCP and CC peaks in the XRD pattern (Figure 3b), which were replaced entirely by HAp peaks, and was further supported by SEM (Figure 5b), which showed clustered HAp structures with no remnants of the OCP and CC structures. Comparative analysis revealed that although both composites promoted HAp formation in SBF, the GelMA-based composite was less efficient in promoting HAp formation than the pig Gel-based composite. The SEM images (Figure 5) highlight this disparity, showing fewer HAp formations in the GelMA-based composite than in the pig-Gel-based composite. This difference can be attributed to variations in the carboxy group content between the two matrices. FTIR analysis (Figure 6b,c) revealed a lower carboxy group content in GelMA (49.8%) compared to pig Gel (60.2%) in the 1750–1475 cm^−1^ range. The carboxy groups serve as critical nucleation sites for HAp; therefore, the reduced carboxy content in GelMA resulted in fewer nucleation sites, leading to less efficient HAp formation. Furthermore, this lower carboxy group content may influence OCP dissolution in SBF, potentially reducing the release of Ca^2+^ and PO_4_^3−^ ions required for HAp growth. In SBF, when the free Ca^2+^ concentration decreases below the supersaturated threshold for OCP but remains supersaturated for HAp, OCP dissolution releases Ca^2+^ and PO_4_^3−^ ions that promote HAp formation post-nucleation. In contrast, the 35% OCP/35% CC/30% pig Gel composite, with its higher carboxy group content due to the crosslinking of pig Gel, chelates more Ca^2+^ ions in SBF, further reducing the Ca^2+^ concentration and promoting more rapid OCP dissolution. This releases additional Ca^2+^ and PO_4_^3−^ ions, thus facilitating HAp growth. Conversely, the lower carboxy group content in the 35% OCP/35% CC/30% GelMA composite may not sufficiently drive this process, resulting in reduced HAp formation compared to the pig Gel-based composite. Such a finding indicates a lower carboxy group content in GelMA than in pig Gel, potentially influencing their respective capacities for HAp nucleation. To overcome this limitation, chemical surface modification with citrate could be a promising approach to improve the HAp formation capacity of OCP/CC/GelMA. Specifically, by immersing the OCP/CC/GelMA composite in a citrate solution, citrate ions are expected to adsorb onto the GelMA surface, potentially chelating calcium ions to form a positively charged calcium–citrate complex. This complex may attract phosphate and additional citrate ions, forming critical-sized clusters that serve as HAp nucleation sites. This strategy is highly promising for increasing the nucleation sites, thus improving HAp formation and the overall bioactivity of the GelMA matrix.

Despite its lower HAp nucleation efficiency in SBF, the GelMA-based composite retains its ability to form HAp, indicating its potential to bond with living bone through the HAp layer. Regardless of a moderate reduction in HAp formation, this capability is more important than the quantity produced, ensuring the potential of the biomaterial for osteoconductivity. The impact of reduced HAp nucleation on bone healing cannot be fully evaluated through SBF immersion tests alone; therefore, further in vitro cellular studies and in vivo experiments are planned to clarify the clinical implications. Importantly, we consider this moderate reduction a non-critical drawback, given the demonstrated capability of the composite to form HAp. Conversely, the GelMA-based composite offers superior initial structural stability in SBF, a feature that is absent in the pig Gel-based composite. This enhanced stability is more valuable for bone regeneration applications, as it prevents rapid in vivo degradation that could impair bone formation and hinder defect repair. Furthermore, the tunable photocrosslinking of the GelMA-based composite enables advanced applications, such as 3D bioprinting, allowing for tailored bone tissue engineering solutions to meet diverse clinical needs. Thus, the GelMA-based composite prioritizes structural integrity and functional performance, significantly outweighing the minor limitation of reduced HAp formation efficiency.

## 4. Materials and Methods

### 4.1. Synthesis of the 35% OCP/35% CC/30% GelMA Composite, 35% OCP/35% CC/30% Pig Gel Composite, Pure GelMA Film, and Pig Gel Film

A 5% GelMA solution was prepared by adding 500 mg of GelMA (PhotoGel^®^-LAP, Methacrylated Gelatin Hydrogel Kit, Sigma-Aldrich, CC324-2, Tokyo, Japan; with a fixed methacrylation degree of >70%, optimized for maximum crosslinking and stiffness) in 9.5 mL of 60 °C PBS followed by shaking at 37 °C using a vortex mixer until fully dissolved. OCP was prepared by heating 100 mL of ultrapure water at 60 °C upon stirring at 500 rpm using a magnetic stirrer, followed by sequential addition of 6.0 mmol of phosphoric acid (H_3_PO_4_, 85% aqueous solution, FUJIFILM Wako Pure Chemical Corporation, Osaka, Japan) and 8.0 mmol of calcium carbonate (CaCO_3_, calcite: 99.5%, Nacalai Tesque, Kyoto, Japan) and stirring for 3 h. The pH of the calcium phosphate slurry was adjusted to 5.0 using hydrochloric acid (HCl, 1 M solution; FUJIFILM Wako Pure Chemical Corporation), followed by an additional 30 min of stirring. The resulting precipitate was collected via vacuum filtration, washed with ultrapure water and ethanol, and dried at 40 °C for a minimum of 12 h. A mixture of OCP and CC powders ([O_2_CCH_2_C(OH)(CO_2_)CH_2_CO_2_]_2_Ca_3_·4H_2_O, 99%, Sigma-Aldrich) was added to the GelMA solution, ensuring that the total mass ratio of the powders (OCP + CC) to GelMA was maintained at 7:3. In the powder phase, OCP and CC were mixed in equal proportions (1:1 mass ratio). This process yielded a slurry with a final composition of 35% OCP, 35% CC, and 30% GelMA. The slurry was subjected to ultrasonic treatment for 20 min to ensure a homogeneous dispersion of the OCP and CC powders. Meanwhile, LAP (PhotoGel^®^-LAP, Methacrylated Gelatin Hydrogel Kit, Sigma-Aldrich, CC324-1) was dissolved in 60 °C PBS to prepare a 17 mg/mL solution. Following ultrasonic dispersion, the 35% OCP/35% CC/30% GelMA slurry was transferred into 24-well plates (300 μL per well) and the LAP photoinitiator solution was immediately added using a micropipette in a darkroom under light-protected conditions to prevent premature activation. The 24-well plate sample size corresponds to the volume exhibiting rapid dissolution in prior OCP/CC/pig Gel experiments, enabling comparison of the performance of GelMA. The LAP solution was introduced in volumetric ratios ranging from 0.02 to 0.1 (*v*/*v*) relative to the GelMA solution. Photo-crosslinking was conducted using a handheld LED light (Elber Co., Ltd., Osaka, Japan), product number 55646, procured from Monotaro, with an emission wavelength of 405 nm. The device features a compact cylindrical design with a diameter of 25 mm and length of 98 mm and is equipped with 21 LED bulbs. During irradiation, the light source was positioned 8 mm above the sample surface and the samples were exposed to 405 nm blue light for varying durations ranging from 1 to 4 min. The gelation state was assessed after each exposure condition to evaluate the crosslinking efficiency. Finally, samples with diameters of 15 mm and thicknesses of 2 mm were obtained. Furthermore, a 5% GelMA solution (2 mL) was prepared separately in a 5 cm diameter plate and subjected to photo-crosslinking for 2.5 min. The cross-linked samples were subsequently immersed twice in ultrapure water for 10 min each, followed by freezing and lyophilization for 48 h, yielding a film-like 5% GelMA structure with a thickness of 0.17 mm.

For comparison, a control group comprising a composite of 35% OCP, 35% CC, and 30% pig Gel was prepared along with a pure pig Gel film. The synthesis process was as follows. The CC powder was blended with the OCP powder in a 1:1 mass ratio to produce a homogeneous OCP/CC ceramic mixture. Pig Gel (Food Grade, Type A, 225 Bloom; MP Biomedicals, LLC, 29525 Fountain Parkway, Solon, OH 44139, USA) was dissolved in ultrapure water at 75 °C to achieve a concentration of 3%. The OCP and CC powders (1:1 mass ratio) were then mixed with the pig Gel solution, maintaining a 7:3 mass ratio of the OCP/CC powders to pig Gel and yielding slurries with a composition of 35% OCP, 35% CC, and 30% pig Gel. The slurries were dispensed into 96-well plates (150 μL per well) and immediately frozen in liquid nitrogen to ensure phase homogeneity. The frozen samples were lyophilized for 48 h. Thermal crosslinking of the OCP/CC/pig Gel composites was performed under vacuum at 150 °C for 24 h. The samples, molded into 96-well plates, exhibited a cylindrical shape with a thickness and diameter of 5 mm. Pure pig Gel samples were also prepared without OCP/CC powders using 2 mL of the pig Gel solution in a 5 cm diameter plate, employing the same lyophilization and thermal crosslinking methods, resulting in a film-like structure with a thickness of 0.14 mm.

### 4.2. Soaking in Simulated Body Fluid

The HAp-forming ability of the samples was evaluated in vitro using SBF [36]. SBF was prepared according to a previously reported method [23]. Subsequently, 35% OCP/35% CC/30% GelMA and 35% OCP/35% CC/30% pig Gel were separately immersed in 30 mL of SBF in individual polypropylene plastic tubes. The tubes were maintained at 37 °C for 7 d, after which the samples were collected and immersed in ultrapure water twice for 10 min each time. The 35% OCP/35% CC/30% GelMA composite samples were frozen and lyophilized for 48 h to obtain freeze-dried samples. The 35% OCP/35% CC/30% pig Gel composite samples were subjected to a 10 min immersion in 99% ethanol, followed by drying in an oven at 40 °C.

### 4.3. Characterization

The initial structural stability in SBF was evaluated by measuring the changes in the diameter of each sample. The crystalline phases were characterized via XRD using a Rigaku MiniFlex 600 diffractometer (Rigaku, Tokyo, Japan) with Cu Kα radiation (λ = 0.154056 nm). The crystal morphology was examined using SEM (JSM-IT200LAJ, JEOL Ltd., Tokyo, Japan) after coating the samples with a thin Au film. The elemental distribution within the samples was further analyzed using EDS coupled with the SEM system. The functional groups of the cross-linked GelMA and pig Gel films were analyzed via FTIR spectroscopy using an ATR accessory with a ZnSe prism (FT/IR-6200, JASCO Corp., Tokyo, Japan; PRO 410-M, JASCO Corp., Tokyo, Japan). Origin 2025 (Student Version) was used for the peak fitting and separation analyses.

## 5. Conclusions

This study contributes to the advancement of bone tissue engineering by developing a 35% OCP/35% CC/30% GelMA composite with enhanced properties for bone regeneration. The principal findings are summarized as follows:

1. The composite exhibits superior structural stability in SBF compared to pig Gel-based composites, which degrade rapidly in SBF.
2. Optimized photo-crosslinking with LAP and blue light enables uniform gelation of 35% OCP/35% CC/30% GelMA.
3. The composite sustains HAp formation in SBF, supporting osteoconductivity; however, its nucleation efficiency is lower than that observed in pig Gel composites.
4. The reduced carboxy content in GelMA possibly contributes to the decreased HAp nucleation, providing insights for further material optimization.
5. The enhanced stability, controlled synthesis, and ability to form HAp in SBF position the composite as a promising scaffold material for bone regeneration applications.

These findings highlight the potential of the 35% OCP/35% CC/30% GelMA composite as a functional and tunable biomaterial for bone tissue engineering.

## Figures and Tables

**Table 1:** Effect of the LAP photoinitiator volumetric ratio and blue light exposure on the status of 35% OCP/35% CC/30% GelMA.

Volumetric Ratio (*v*/*v*) of LAP Photoinitiator in GelMA	Blue Light Exposure Time (min)	Sample Status After Blue Light Exposure
0.02	2	Fluid
	4	Fluid
0.06	1	Fluid
	1.5	Fluid
0.08	1	Fluid
	1.5	Upper side gelled Bottom side fluid
0.1	1	Fluid
	1.5	Upper side gelled Bottom side fluid
	2	Upper side gelled Bottom side fluid
	2.5	Both sides gelled

## Data Availability

Data are contained within the article.

## Abbreviations

The following abbreviations are used in this manuscript:

OCP	octacalcium phosphate
CC	calcium citrate
Gel	gelatin
GelMA	methacrylated gelatin
SBF	simulated body fluid
HAp	hydroxyapatite
XRD	X-ray diffraction
SEM	scanning electron microscopy
FTIR	Fourier-transform infrared
ATR	attenuated total reflectance
EDS	energy-dispersive X-ray spectroscopy
